# Green flowers need yellow to get noticed in a green world

**DOI:** 10.1093/aob/mcae213

**Published:** 2024-12-10

**Authors:** José C del Valle, Melissa León-Osper, Carlos Domínguez-González, Mª Luisa Buide, Montserrat Arista, Pedro L Ortiz, Justen B Whittall, Eduardo Narbona

**Affiliations:** Department of Plant Biology and Ecology, Facultad de Biología, Universidad de Sevilla, Seville, Spain; Department of Molecular Biology and Biochemical Engineering, Facultad de Ciencias Experimentales, Universidad Pablo de Olavide, Seville, Spain; Department of Molecular Biology and Biochemical Engineering, Facultad de Ciencias Experimentales, Universidad Pablo de Olavide, Seville, Spain; Department of Molecular Biology and Biochemical Engineering, Facultad de Ciencias Experimentales, Universidad Pablo de Olavide, Seville, Spain; Department of Plant Biology and Ecology, Facultad de Biología, Universidad de Sevilla, Seville, Spain; Department of Plant Biology and Ecology, Facultad de Biología, Universidad de Sevilla, Seville, Spain; Department of Biology, Santa Clara University, Santa Clara, CA, USA; Department of Molecular Biology and Biochemical Engineering, Facultad de Ciencias Experimentales, Universidad Pablo de Olavide, Seville, Spain

**Keywords:** Chromatic contrast, flower conspicuousness, green flowers, plant–pollinator interaction, reflectance spectra, visual modelling, yellowish hue

## Abstract

**Background and Aims:**

Flower colour is a key feature in plant–pollinator interactions that makes the flowers visible amid the surrounding green vegetation. Green flowers are expected to be scarcely conspicuous to pollinators; however, many of them are visited by pollinators even in the absence of other traits that might attract pollinators (e.g. floral scents). In this study, we investigate how entomophilous species with green flowers are perceived by pollinators.

**Methods:**

We obtained reflectance spectra data of 30 European species that display green or green–yellow flowers to the human eye. These data were used to perform spectral analyses, to calculate both chromatic (colour contrast against the background) and achromatic (colour contrast that relies on the signals from the green-sensitive photoreceptors) cues and to model colour perception by hymenopterans (bees) and dipterans (flies).

**Key Results:**

The visibility of green flowers to bees and flies (i.e. their chromatic contrast values) was lower compared with other floral colours commonly pollinated by these insects, whereas green–yellow flowers were as conspicuous as the other flower colours. Green flowers with low chromatic contrast values exhibited higher achromatic contrast, which is used to detect distant flowers at narrow visual angles, than green–yellow flowers. Additionally, the marker points (i.e. sharp transition in floral reflectance that aids pollinators in locating them) of green and green–yellow flowers aligned to some degree with the colour discrimination abilities of bees and flies.

**Conclusions:**

We found that many entomophilous green and green–yellow flowers are conspicuous to bees and flies through their chromatic or achromatic contrasts. Although acquiring pigments such as carotenoids, which impart a yellowish hue to flowers and enhance their visibility to pollinators, could increase their conspicuousness, the metabolic costs of pigment production, along with the use of alternative strategies to attract pollinators, might have constrained carotenoid emergence in certain lineages of green-flowered species.

## INTRODUCTION

Colour plays a key role in ecological interactions and evolutionary processes. Conspicuous colours and ornamentations are a widespread phenomenon in the animal kingdom that can function in different ways during animal communication (Endler, 1978, 1992; Stevens, 2013). Extravagant and colourful ornaments in males are hypothesized to signal the overall individual quality to females, obtaining greater fitness benefits through multiple mating (Darwin, 1871; but see Nolazco *et al*., 2022). Aposematic animals are brightly coloured to advertise that they are unprofitable to potential predators, whereas cryptic species use coloration to blend in with their environments and avoid detection (Strauss and Cacho, 2013; Cuthill *et al.*, 2017). In plants, colour serves as a vital cue in mutualist interactions, such as pollination or seed dispersal (Willson and Whelan, 1990; Stournaras *et al.*, 2013). Flower colour is one of the main components of plant–pollinator signalling, crucial for enhancing flower visibility against the surrounding background and for drawing the attention of pollinators (Van Der Kooi *et al.*, 2019). Flowers that maximize contrast with the natural background, most often composed of green foliage, are more easily detected by pollinators and can influence their foraging choices (De Ibarra *et al.*, 2015; Bukovac *et al.*, 2017; Finnell and Koski, 2021).

Green flowers, as perceived by humans, are relatively rare in nature, representing approximately <10 % of species in different regions around the world (Weevers, 1952; Utech and Kawano, 1975; Dyer *et al.*, 2021; Wang *et al.*, 2022). These flowers are commonly associated with wind pollination owing to the absence of striking floral pigments that attract pollinators (Faegri and van der Pijl, 1979; Fenster *et al.*, 2004; Friedman and Barret, 2009; Rosas-Guerrero *et al.*, 2014). Dyer *et al.* (2021) reported that ~45 % of the total European flora that are pollinated abiotically (e.g. wind or water pollination) displayed green flowers. Likewise, historical records of the flora from eastern North America showed that 82 % of native green-flowered species were wind pollinated, while small and sometimes imperfect, entomophilous green flowers were sparingly visited by non-specialized insects and were also self-compatible (Lovell, 1912). A likely explanation for the rarity of pollinator-dependent species exhibiting green flowers is the expected low contrast they create against the predominantly green background vegetation (Chittka *et al.*, 1994; Moré *et al.*, 2020). In fact, many greenish and brownish flower colours are inconspicuous to flower herbivores, thus protecting plants from florivory (Kemp and Ellis, 2019). However, some species that display green or greenish flowers are successful in attracting pollinators (Herrera *et al.*, 2001; Alonso, 2005). Some of these flowers produce floral scents to attract pollinators, such as in the case of two African *Eucomis* species, whose green flowers emit aromatic and monoterpene scents that attract pompilid wasps (Shuttleworth and Johnson, 2009). Hence, a specialized pollination syndrome has been defined that combines cryptic green flowers with scents as floral attractants (Johnson *et al.*, 2007; Brodmann *et al.*, 2008). However, other species that display green or greenish flowers appear to be odourless, at least to humans, such as *Daphne laureola* and several species of *Euphorbia*, yet they are visited by generalist pollinators (Herrera *et al.*, 2001; Narbona *et al.*, 2002; Alonso, 2005; Asenbaum *et al.*, 2021). The flowers of these species are visited mainly by non-specialized pollinators from the orders Hymenoptera and Diptera (Fenster *et al.*, 2004; Lukas *et al.*, 2020; Asenbaum *et al.*, 2021). Although rare, bird pollination has also been documented in some other green-flowered species (Faegri and van der Pijl, 1979; Rebelo and Siegfried, 1985; Rathcke, 2000; Mizuno *et al.*, 2024).

Although abiotic factors might influence flower colour evolution, this trait is shaped primarily by biotic selection pressures, with pollinators acting as key agents and driving floral reflectance to align with their visual sensitivities (Fenster *et al.*, 2004; Shrestha *et al.*, 2013b; Dyer *et al.*, 2021; Dorin *et al.*, 2023). In this context, it is crucial to determine how green flowers are perceived visually by their most common pollinators, namely hymenopterans and dipterans (Supplementary Data Table S1). Regarding hymenopterans, the honeybee *Apis mellifera* is used as a model organism for studies on colour vision (Van Der Kooi *et al.*, 2021). Honeybees and other hymenopterans are trichromatic, having three photoreceptors, with peak sensitivities in the ultraviolet (UV), blue and green regions of the light spectrum (Peitsch *et al.*, 1992; Briscoe and Chittka, 2001; Chittka and Raine, 2006; Dyer *et al.*, 2008). Dipterans are another important group of pollinators, although their visual system has received less attention (Garcia *et al.*, 2022). Blowflies and hoverflies typically have four sensitivity peaks covering the UV, blue and green–yellow wavelengths (Troje, 1993; An *et al.*, 2018; Hannah *et al.*, 2019), although spectral sensitivity varies widely between species (reviewed by Van Der Kooi *et al.*, 2021). Therefore, the visual systems of pollinating bees and flies, both sensitive to the green region of the visible spectrum, suggest that they can perceive green flowers. In addition, the visual system of pollinators demonstrates optimal colour discrimination in specific regions of the light spectrum, typically aligning with the wavelength range where the sensitivity of two photoreceptors overlaps (Chittka and Menzel, 1992; Shrestha *et al.*, 2013b). Yet, the reflectance spectra of flowers can display areas characterized by abrupt transitions (referred to as marker points), indicating sharp changes in the spectrum (Shrestha *et al*., 2013). If marker points align with the wavelengths associated with optimal discrimination ability of a pollinator group, it might indicate an adaptation of the floral colour to the visual system of the animal (Dyer *et al.*, 2012; Shrestha *et al.*, 2013a, 2016; Camargo *et al.*, 2019). Thus, bees show their maximum discrimination wavelengths at 400 and 500 nm (Chittka and Menzel, 1992; Shrestha *et al.*, 2013a), whereas the wavelengths of maximum discrimination for flies have not yet been studied.

In addition to the ability of pollinators to perceive flower colours, foraging relies on the contrast between the floral stimulus and its background (i.e. chromatic contrast), typically green foliage, and this contrast aids in flower detection, attracting pollinators to the food source (Menzel and Backhaus, 1991). For bees, this parameter is crucial, because they use chromatic signals to detect a target when foraging in short distances (Giurfa *et al.*, 1996), and it is likely to play a key role in stimulus detection in flies (An *et al.*, 2018). Additionally, achromatic contrast (i.e. contrast based on signals from green-sensitive photoreceptors) has been suggested to be important in detection and discrimination tasks in bees, because they rely on this parameter when foraging from long distances (Spaethe *et al.*, 2001). However, achromatic contrast is less well characterized in flies (van der Kooi and Kelber, 2022). For green flowers, the degree of conspicuousness (i.e. the chromatic and achromatic contrasts) of these flowers against a background of green foliage has not yet been quantified, nor has their attractiveness to pollinators been evaluated relative to other floral colours.

The goal of this study was to assess how flowers that appear green or greenish to human eyes are perceived by pollinators. We determined whether these flowers are inconspicuous, as traditionally thought based on human perception, or whether, after accounting for pollinator visual systems, they are more conspicuous than expected. Specifically, we analysed the reflectance spectra of 30 entomophilous species that display green flowers found in Europe, where hymenopterans (bees) and dipterans (flies) constitute the primary pollinator groups (Ashworth *et al.*, 2015). Although green flowers are rare, greenish-yellow flowers are more common, hence we chose to include both groups and compare their conspicuousness to pollinators in contrast to their backgrounds. We examined the chromatic and achromatic contrasts of these flowers according to the vision models of their main pollinators and compared their conspicuousness with other flower colours. Furthermore, the position of the marker points in the reflectance spectra of the green and green–yellow flowers were compared with the spectral discrimination sensitivities of bees to assess the correspondence between reflectance spectra of these flowers and the colour discrimination ability of this pollinator (Chittka and Menzel, 1992; Shrestha *et al.*, 2013a). We specifically addressed the following questions. What is the perception by the main pollinators (i.e. bees and flies) based on their visual systems, of colour stimuli generated by green and green–yellow flowers? Do green and green–yellow flowers have higher or lower chromatic and achromatic contrast than flowers of other colours in the visual systems of bees and flies? Are green and green–yellow flowers equally visible to these pollinators? Do the spectral characteristics of these flowers align with the colour discrimination abilities of pollinators? All this information would illuminate whether green and green–yellow flowers are similarly conspicuous to their pollinators.

## MATERIALS AND METHODS

### Study species

**Fig. 1.**
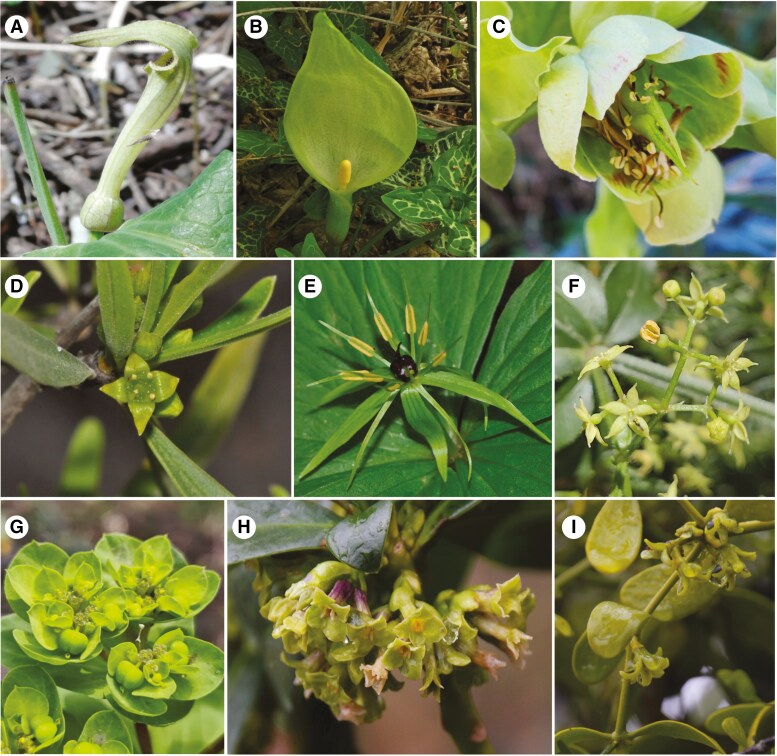
Pictures of some of the species displaying green (A–G) or green–yellow (H, I) flowers included in this study: (A) *Aristolochia paucinervis* (Aristolochiaceae); (B) *Arum italicum* (Araceae); (C) *Helleborus foetidus* (Ranunculaceae); (D) *Rhamnus lycioides* (Rhamnaceae); (E) *Paris quadrifolia* (Melanthiaceae); (F) *Rubia peregrina* (Rubiaceae); (G) *Euphorbia nicaeensis* (Euphorbiaceae); (H) *Daphne laureola* (Thymelaeaceae); and (I) *Viscum cruciatum* (Santalaceae).

We studied 30 entomophilous European species that display green flowers as perceived by the human eye, although some of them have a yellowish hue in human vision (we refer to them as ‘green–yellow flowers’ hereafter). The spectrum of all flowers generally showed very low reflectance values in the UV (300–400 nm) and blue (400–500 nm) regions of the visible (VIS) spectrum, with a primary reflectance peak occurring in the green (500–550 nm) region of the VIS light, which then decreased until reaching a valley at ~670 nm (Supplementary Data Fig. S1). A principal component analysis using reflectance data from 300 to 700 nm and colour as a grouping variable (i.e. green and green–yellow) was conducted to confirm the validity of the separation between green and green–yellow flowers based on human colour perception (Supplementary Data Fig. S2). Examples of species displaying green flowers and those with a yellowish coloration include *Rhamnus lycioides* and *Daphne laureola*, respectively (for additional examples, see Fig. 1).

### Floral reflectance data

For 19 of the 30 species, we conducted measurements of naturally growing flowers in the field from populations located in southern Spain (Supplementary Data Table S1). We acquired the floral reflectance data in the laboratory from flowers collected in the field using a Jaz portable spectrometer equipped with a deuterium–tungsten halogen light source (Ocean Optics, Dunedin, FL, USA) and a black metal probe holder (6 mm diameter opening at 45°). We measured reflectance curves from 300–700 nm wavelengths at 0.4 nm intervals, setting an integration time of 2 s and smoothing boxcar width of 12 (Del Valle *et al.*, 2018). Reflectance measurements were calibrated with a white standard (WS-1-SL, Ocean Optics) and analysed using both Spectra Suite v.10.7.1 and OceanView v.2.0 software. For each species, we selected between one and ten flowers (always using a single flower per plant) to measure the reflectance of petals. Alternatively, we measured the reflectance of bracts, spathes or the larger floral structures of an inflorescence that could attract pollinators (for further details, see Supplementary Data Table S1). We used the ‘*aggplot*’ function of the *pavo* R package (Maia *et al.*, 2019) to aggregate all the spectra from the same species, then to obtain the average and standard deviation (s.d.) values. For the remaining 11 species, we downloaded floral reflectance data (one spectrum per species; Supplementary Data Table S1) from the Floral Reflectance Database (http://www.reflectance.co.uk/; hereafter ‘FReD’) (Armold *et al.*, 2010). We conducted a search in FReD, filtering for the plant attribute ‘Human Colour: green’ (accessed 25 November 2021). We considered only those entries located in Europe and the Eastern Mediterranean. The results obtained were validated with a subsequent bibliographical search to confirm that the floral coloration of these species was predominantly or entirely human green. Data from both direct measurements and FReD were processed equally, as described in the next section, to be used in spectral analyses.

### Spectral analysis

For reflectance spectra analyses, we considered wavelengths between 300 and 700 nm (Briscoe and Chittka, 2001; Endler and Mielke, 2005). We used the ‘*procspec*’ function of the *pavo* R package (Maia *et al.*, 2019) to process reflectance curves. We smoothed reflectance curves with a smoothness parameter of 0.20. Subsequently, we removed negative values from four samples to correct minor deviations during calibrations by setting the minimum value to zero and scaling other values accordingly (White *et al.*, 2015).

### Perception of green and green–yellow flowers by pollinators

We used animal vision models to represent how a colour stimulus is perceived by the two main functional groups of pollinators considered in this study: hymenopterans (bees) and dipterans (flies). We plotted the processed reflectance curves as loci in the vision models of both pollinator groups using the function ‘*colspace*’ in the *pavo* R package (Maia *et al.*, 2019), as described by Narbona *et al.*, (2021a). For bees, the most widely used vision model is the so-called ‘colour hexagon’ of the honeybee *A. mellifera*, which represents a continuous spectrum of colour perception, encompassing UV, UV–blue, blue, blue–green, green and UV–green regions (Chittka, 1992; Chittka and Wells, 2004; Chittka and Kevan, 2005). For flies, we used the space model for the tetravariant visual system of *Lucilia* sp. developed by Troje (1993). This colour space has four quadrants that represent a continuous spectrum of colours, including UV, purple, blue and yellow (Troje, 1993; Lunau, 2014). Although bee and fly colour vision models are categorical, recent studies demonstrate that colour discrimination of both groups of insects could be continuous (Garcia *et al.*, 2017, 2022; Hannah *et al.*, 2019). We implemented von Kries transformation for both models, which assumes that eye photoreceptors are adapted to the spectral properties of the illumination and background (White *et al.*, 2015). We used the standard daylight function (D65 irradiance function) as illuminant and the average spectrum of green foliage of 230 species proposed by Chittka (1992) as background in the vision models, which is the most commonly used set-up in similar studies (Chen *et al.*, 2020; Dyer *et al.*, 2021; Narbona *et al.*, 2021a; León-Osper and Narbona, 2022). For the specific case of bees, we considered a hyperbolic transformed quantum catch. We obtained the chromatic contrast against the background, i.e. the distance between the colour loci of the flower and the achromatic centre measured in Euclidean units (hereafter, EU), according to the photoreceptor spectral sensitivities of the two colour space models (Rohde *et al.*, 2013; Hannah *et al.*, 2019; Garcia *et al.*, 2022). Additionally, we considered the empirically determined discriminability thresholds of 0.11 EU for *A. mellifera* and 0.096 EU for *Eristalis tenax* (putatively extendable to other hymenopterans and dipterans, respectively) as an estimate of whether a colour stimulus is easily discriminated from the background (Dyer *et al.*, 2012; Garcia *et al.*, 2022). It should be noted that these thresholds must be handled with caution, because they were established in laboratory conditions and do not consider additional factors that might significantly impact the chromatic contrast of the flower, such as the surrounding background or the presence of other nearby stimuli (Giurfa *et al.*, 1997; Bukovac *et al.*, 2017). For bees, we also calculated the achromatic contrast (i.e. green contrast), which is the modulation of the green receptor against the background and is calculated as the excitation value of the green photoreceptor different from 0.5, which represents adaptation to the background (Spaethe *et al.*, 2001).

### Flower colour conspicuousness in relationship to other flower colours

To compare the conspicuousness (i.e. chromatic and achromatic contrasts) of green (*n* = 19) and green–yellow (*n* = 11) flowers relative to more common flower colours in nature, we used a dataset comprising 100 species categorized into four colour groups based on human perception of their flowers: yellow (*n* = 38), blue–violet (*n* = 28), pink (*n* = 25) and white (*n* = 9) (Supplementary Data Fig. S3). The reflectance data for non-green flowers were sourced from a previous study on flower colour perception considering the visual systems of the main groups of pollinators present in the Mediterranean Basin (Narbona *et al.*, 2021a).

### Spectral signals: marker points

To identify marker points of green and green–yellow flowers in the UV–VIS range (300–700 nm), we used the software Spectral-MP developed by Dorin *et al.* (2020) with the recommended settings: changes in reflectance of ≥10 % occurring over a wavelength range of 50 nm with a smoothing window of ±10 data points and considering five data points to look ahead when performing slope change detection. We also calculated the frequency of marker points in wavelength bins of 10 nm across the UV–VIS reflectance spectra for all species. Then, we measured the matching between detected marker points and the wavelengths of maximum discrimination of bees. To do so, we calculated two metrics: the mean absolute deviation (MAD) and the minimal absolute deviation (minAD) (Shrestha *et al.*, 2013a). MAD measures the average proximity of flower marker points to each wavelength of maximum discrimination, and minAD measures the minimum distance between marker points and each specific wavelength of optimal visual discrimination (for bees, we thus obtain one MAD value and two minAD values: minAD_400_ and minAD_500_) (Shrestha *et al.*, 2013a). For both metrics, the smaller values imply a closer fit between floral reflectance spectra to a particular visual system. For comparison, we also identified the marker points and calculated the MAD and minAD spectral metrics for the 28 blue–violet flowers considered in this study. This comparison was performed considering that bees have an innate preference for blue and violet flowers (Giurfa *et al.*, 1995).

### Statistical analyses

Differences in conspicuousness (i.e. achromatic and chromatic contrasts) between green flowers and other flower colour categories, and between green and green–yellow flowers, were analysed using phylANOVAs (Garland *et al.*, 1993). This approach controls for the potential influence of phylogeny when analysing differences among flower colour groups. Initially, we built a phylogenetic tree using the ‘*phylo.maker*’ function implemented in the *V.PhyloMaker* R package (Jin and Qian, 2019), which relies on the angiosperm megatree as a phylogenetic backbone (Zanne *et al.*, 2014; Smith and Brown, 2018). Then, we performed phylANOVAs using the *phytools* R package (Revell, 2012), with 10 000 simulations for each test and Holm-adjusted *P*-values for *post hoc* comparisons. PhylANOVAs were also used to test for differences in the MAD and minAD parameters between green, green–yellow and blue–violet flowers. The principal component analysis used to assess the suitability of grouping the species into green or green–yellow categories was performed using the R package *factoextra* (Kassambara and Mundt, 2020). We used RStudio v.2022.12.0 (RStudio Team, 2022) to perform all statistical analyses.

## RESULTS

### Pollinators’ perception

**Fig. 2.**
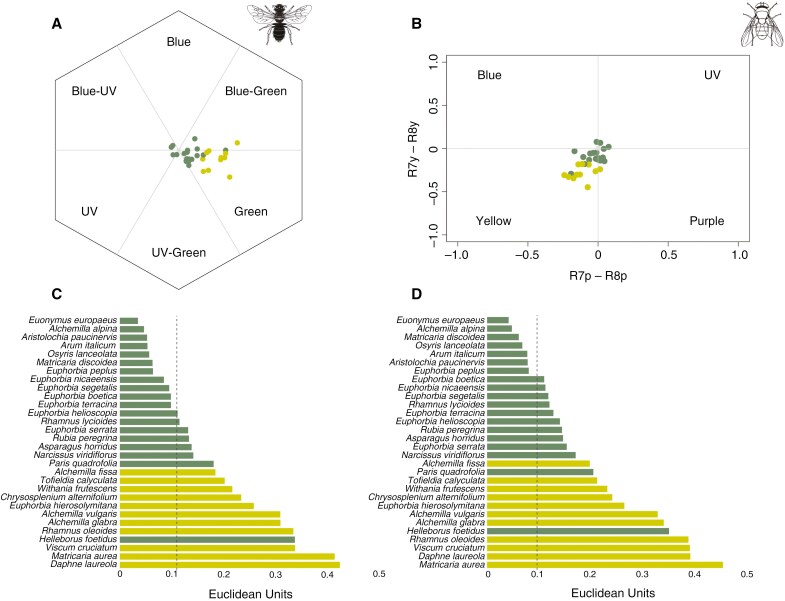
(A, B) The colour loci of the 30 species that display green (dark green dots) or green–yellow (lime green dots) flowers are represented in the hexagon colour space of bees (A) and the tetravariant visual system of flies (B). (C, D) The chromatic contrast values of the 30 species that display green (dark green bars) or green–yellow (lime green bars) flowers, obtained in the pollinator visual systems of bees (C) and flies (D), are shown. Species have been sorted by increasing values obtained from each vision model. Chromatic contrast values are expressed in Euclidean units (EU). The dotted lines represent the theoretical discriminability threshold for hymenopteran and dipteran vision models (0.11 and 0.096 EU, respectively). Animal silhouettes were taken from Divulgare (http://divulgare.net/) under a Creative Commons licence.

For the bee vision, most green (13 of 19) and green–yellow (9 of 11) flowers occupied the green category in the hexagon colour space (Fig. 2A; Supplementary Data Table S2). The chromatic contrast of the green flowers was variable, ranging from 0.035 to 0.338 EU, with a mean ± s.d. of 0.107 ± 0.068 EU (Fig. 2C), and the mean of green–yellow flowers was significantly higher (0.293 ± 0.082 EU; phylANOVA: *F*_1,29_ = 44.8, *P* < 0.001), with values ranging between 0.184 and 0.424 EU. Eleven of the 19 green flowers showed a chromatic contrast of <0.11 EU, the theoretical discriminability threshold for bees, whereas none of the green–yellow flowers did (Fig. 2C; Supplementary Data Table S2). These data suggest that the green flowers are, presumably, more difficult for bees to perceive. When examining the achromatic contrast of green flowers, the mean was 0.176 ± 0.089 EU (range = 0.036–0.322 EU), which was statistically higher than green–yellow flowers (mean = 0.102 ± 0.073 EU; range = 0.001–0.200 EU; phylANOVA for specific comparison green vs. green–yellow flowers: *F*_1,29_ = 5.51, *P* = 0.045; Supplementary Data Fig. S4; Table S2).

For the fly vision, green flowers concentrated the majority of colour loci in the yellow and the purple sectors (10 and 6 of 19, respectively), whereas all green–yellow flowers except one occupied the yellow section (Fig. 2B; Supplementary Data Table S2). The chromatic contrast was variable, ranging from 0.041 to 0.350 EU for green flowers, with a mean ± s.d. of 0.124 ± 0.070 EU. The chromatic contrast values obtained from green–yellow flowers was significantly higher than for green flowers (0.312 ± 0.087 EU; phylANOVA: *F*_1,29_ = 42.3, *P* < 0.001), ranging between 0.197 and 0.453 EU. Seven of the 19 green flowers and none of the green–yellow flowers were below the theoretical discriminability threshold for flies (0.096 EU; Fig. 2D; Supplementary Data Table S2), suggesting that they might be more difficult for flies to perceive. As indicated above, achromatic contrast is not available for the fly visual system because it is not yet well defined.

### Conspicuousness of green and green–yellow flowers compared with other flower colours

**Fig. 3.**
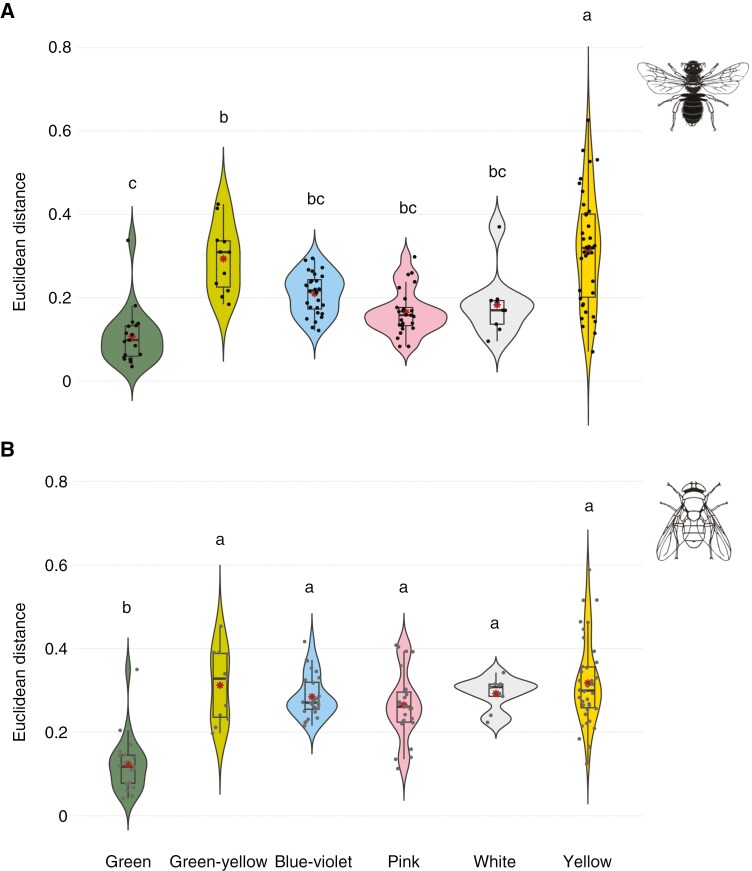
Violin plots with boxplots represent the distribution of chromatic contrast values, expressed in Euclidean units (EU), obtained from the vision model of bees (A) and flies (B). The colour of each violin plot corresponds to the six flower colour categories considered: green (*n* = 19), green–yellow (*n* = 11), blue–violet (*n* = 28), pink (*n* = 25), white (*n* = 9) and yellow (*n* = 38). Boxplots represent the interquartile range, the thin black line depicts 1.5 × interquartile range, the central line displays the median, and the red and small black points represent the mean and all sample values, respectively. Violin plots show the full distribution of sample values for each flower colour category. Different letters indicate significant differences at the 0.05 level. Animal silhouettes were taken from Divulgare (http://divulgare.net/) under a Creative Commons licence.

The phylogenetic ANOVA revealed significant differences among flower colours regarding their visibility (i.e. chromatic contrast) to bees (phylANOVA: *F*_5,129_ = 18.02, *P* < 0.001) and to flies (phylANOVA: *F*_5,129_ = 16.52, *P* < 0.001). For both bees and flies, green flowers showed the lowest chromatic contrast values. For bees, the chromatic contrast of green flowers did not differ from those of blue–violet, pink and white flowers (Fig. 3A), although this was not the case for flies (Fig. 3B). Green–yellow flowers exhibited significant higher conspicuousness than green flowers for both bees and flies (Fig. 3). For bees, we also found significant differences among flower colours when analysing the contrast to the green receptor of bees (i.e. achromatic contrast; phylANOVA: *F*_5,129_ = 23.57, *P* < 0.001). Achromatic contrast values were statistically greater in green relative to green–yellow flowers, and white flowers had the highest achromatic contrast values (Supplementary Data Fig. S5).

### Analysis of marker points

**Fig. 4.**
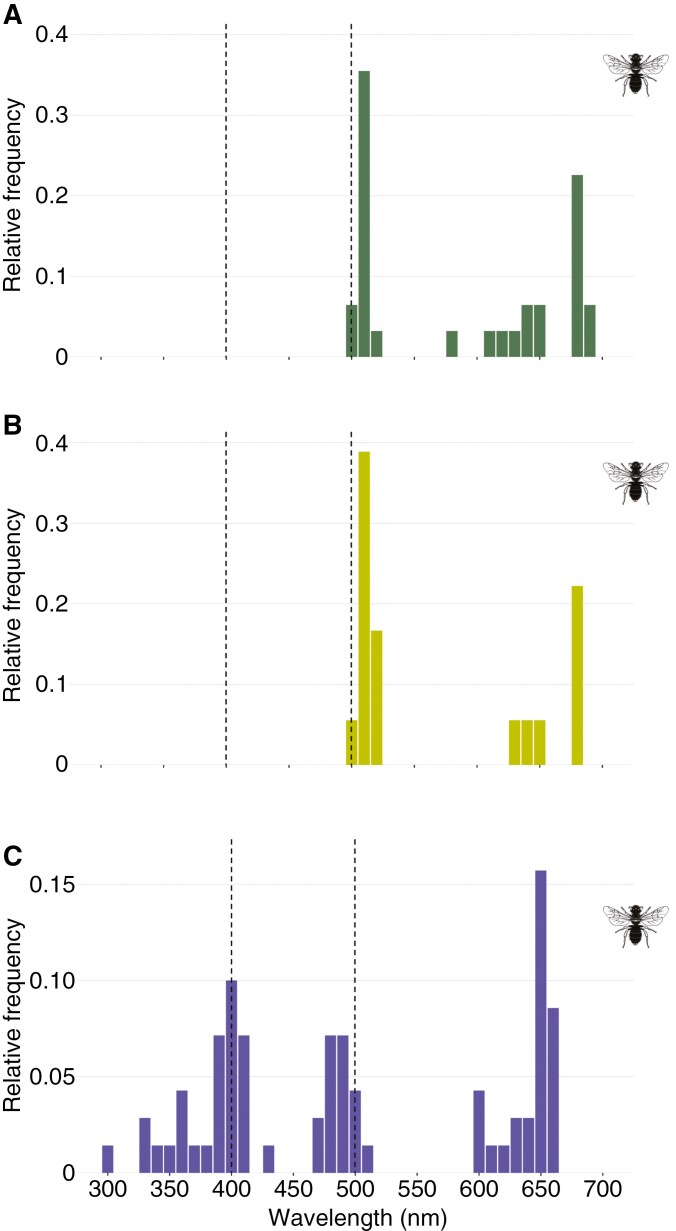
Relative frequency distribution of marker points found in species displaying green (A), green–yellow (B) and blue–violet (C) flowers. Dotted lines at 400 and 500 nm indicate the hue-discrimination optima of the hymenopteran visual system. Animal silhouettes were taken from Divulgare (http://divulgare.net/) under a Creative Commons licence.

Four of the 30 studied species (13 %) presented reflectance spectra that did not generate marker points with the recommended settings (Supplementary Data Fig. S6). Green flowers exhibited most marker points clustered near 500 nm (45.2 % of inflection points were in the 480–520 nm range; Fig. 4A) and 600 nm (54.8 %). Green–yellow flowers, in contrast, concentrated a higher proportion of marker points at 500 nm (61.1 %), with the remaining found at 600 nm (38.9 %) (Fig. 4B). When analysing marker points in the reflectance spectra of blue–violet flowers, we found an aggregation around 500 nm (22.9 % in the 480–520 nm range) and at wavelengths of >600 nm (37.1 %). However, in this case, marker points were also concentrated near 400 nm (27.1 % in the 380–420 nm range) or even within the UV range (12.9 % in the 300–380 nm; Fig. 4C).

When comparing MAD and minAD values among green, green–yellow and blue–violet flowers, we found no statistical differences for the MAD and minAD_500_ parameters (Table 1). However, minAD_400_ differed significantly among floral colours (phylANOVA: *F*_2,57_ = 91.1, *P* = 0.001; Table 1). Blue–violet flowers demonstrated a significantly better match for optimal bee discrimination at 400 nm, with values nearly 6-fold lower (closer to the bee optimal) than those obtained for the green and green–yellow flowers.

**Table 1.** Comparisons of mean absolute deviation (MAD) and minimal absolute deviation (minAD) values for hymenopterans between green, green–yellow and blue–violet flowers. Mean ± s.d. values for MAD and minAD parameters are shown; ‘ns’ indicates a non-significant *P*-value.

Parameter	Green flowers	Green–yellow flowers	Blue–violet flowers	Significance
MAD Hymenoptera	96.5 ± 57.4	70.8 ± 48.5	77.0 ± 15.8	ns
minAD_400_	125.3 ± 45.5	116.5 ± 4.9	19.9 ± 19.1	0.001
minAD_500_	25.3 ± 45.5	16.5 ± 4.9	42.0 ± 41.4	ns

## DISCUSSION

Our results suggest that a considerable number of species with green flowers in Europe, particularly those with a green–yellow hue, appear to be detectable visually by hymenopterans and dipterans, the two most important pollinator groups in this region. Indeed, green–yellow flowers were as conspicuous as other flower colours commonly visited by both groups of pollinators. Although the canonical view is that green flowers lack both chromatic and achromatic cues, we demonstrated that the spectral characteristics of green and green–yellow flowers enable many of them, particularly the green–yellow ones, to be distinguished visually from the surrounding green background. This distinctiveness renders them conspicuous to both bees and flies. Below, we discuss the potential adaptive significance of green and green–yellow flowers with respect to the visual systems of their pollinators.

### Not all green flowers are equally conspicuous to bees and flies

Green flowers were less visible to pollinators, on average, in comparison to the green–yellow ones, which were nearly three times more conspicuous. Approximately 58 % of the pure green-flowered species studied here showed chromatic contrast values lower than the theoretical discriminability threshold for bees, and 37 % showed values lower than the threshold for flies. This suggests that these flowers might be more challenging for these insects to distinguish from the background. Green flowers showed lower chromatic contrast than some of the other flower colours, although adding a yellowish hue enhanced their visibility to both bees and flies. For bees, green flowers showed lower chromatic contrast than green–yellow and yellow flowers but not blue, white or pink flowers. For flies, green flowers showed lower chromatic contrast relative to all other flower colours tested. As far as we know, green and green–yellow flowers have not been included explicitly in previous studies analysing chromatic contrast in the visual systems of honeybees and flies. The mean chromatic contrast values for other typical bee-pollinated flower colours were comparable to those observed in green and green–yellow flowers. For instance, Coimbra *et al.* (2020) found that the average chromatic contrast for bee-pollinated flowers across >200 species worldwide ranged from 0.13 EU for pink flowers to 0.22 EU for white flowers. This range of values encompasses those we found for approximately one-third of green flowers and for all green–yellow flowers (0.131–0.424 EU). Likewise, in another study on bee-pollinated species from the Mediterranean Basin displaying different flower colours, the mean chromatic contrast value was 0.23 EU, slightly lower than that of the green–yellow flowers presented here (0.293 EU on average) (Ortiz *et al.*, 2021). Our results suggest that certain species with green flowers, particularly when combined with yellow hues, might be as conspicuous to bees as other flower colours, at least based on their chromatic contrast. For flies, however, green flowers would be likely to benefit from the addition of yellow pigmentation to enhance their visual attraction. Although the perceived chromatic contrast between a flower and its surroundings plays an important role in flower detection and recognition by pollinators (Giurfa *et al.*, 1996; Kelber *et al.*, 2003; Dyer and Chittka, 2004; van der Kooi *et al.*, 2019), it is important to note that chromatic contrast is only one of several factors that influence pollinator preferences. Attributes such as floral colour, flower size, scent, rewards and pollinator learning also play significant roles, because colour represents only one component of the multisensory cues that guide pollinator foraging.

The comparison of marker points among green, green–yellow and blue–violet flowers revealed both differences and similarities. All three flower colours showed a significant number of marker points in the red region (600–700 nm) of the light spectrum. This region lacks relevance for bees owing to their long-wavelength photoreceptors having low sensitivity at these wavelengths (Chittka and Wells, 2004), but probably enhances detection by other insects that might have sensitivities close to these longer wavelengths. Typically, bee-pollinated flowers show a high number of marker points in two regions of the light spectrum, around 400 nm (violet–blue) and 500 nm (blue–green) (Giurfa *et al.*, 1995; Dyer *et al.*, 2012; Bischoff *et al.*, 2013), where the visual system of bees is maximally sensitive (Chittka and Menzel, 1992). In our study, blue–violet flowers had marker points in these two regions of the light spectrum, suggesting integration of this flower colour with the visual system of bees (Shrestha *et al.*, 2013a). Conversely, green and green–yellow flowers clustered their spectral marker points primarily around 500 nm, consistent with the statistical differences found in the comparison of minAD_400_ but not in the case of the minAD_500_ spectral metric. Thus, the spectral signatures of green flowers would activate only one region of maximum discrimination at 500 nm, as opposed to the two regions in blue flowers (400 and 500 nm). Moreover, bees have an innate, precise ability to discriminate colours around 400 nm (Menzel, 1967; Giurfa *et al.*, 1995; Chittka and Wells, 2004). The implications of this are not entirely clear. Although it is true that flower-naïve bees frequently prefer blue–violet and green colours, their innate preference for the former is linked to the higher nectar rewards associated with these flowers (Giurfa *et al.*, 1995). This preference is thought to be inherited, reflecting a phylogenetically ancient tendency (Chittka and Wells, 2004).

Unlike bees, flies typically prefer flowers with higher reflectance at longer wavelengths, especially yellow flowers (560–580 nm) (Briscoe and Chittka, 2001; Lunau, 2014; Hannah *et al.*, 2019). Shrestha *et al.* (2019) found that, contrary to bee-pollinated orchids, fly-pollinated ones present their marker points at longer wavelengths, but never at wavelengths of <500 nm. Likewise, the floral spectra of native flora from the Macquarie Islands, consisting of pale cream–green flowers exclusively pollinated by flies, were characterized by spectral marker points predominantly occurring around 510 and 690 nm, occupying the yellow quadrant in the colour vision system of flies (Shrestha *et al.*, 2016). Thus, the concentration of marker points in long wavelengths (500–700 nm) of European species with green and green–yellow flowers might, potentially, facilitate colour discrimination by flies, at least for syrphid flies (An *et al.*, 2018; Hannah *et al.*, 2019). In addition, a substantial proportion of the species studied here clustered in the yellow region of the colour space model for the fly vision, especially among green–yellow flowers, which is consistent with the preference of flies for long-wavelength-rich, ‘yellow’ coloration (Lunau, 1990; Shrestha *et al.*, 2016, 2019; An *et al.*, 2018). Finally, the low UV reflectance of green–yellow flowers is also in line with the innate preferences of generalist dipterans for yellow colours without UV reflection, probably owing to the effects of UV wavelengths on flower brightness and colour saturation, which influences colour choice in flies (An *et al.*, 2018). Our findings suggest that the spectral signatures of most green and green–yellow flowers, including their marker points, appear to be adjusted, to some extent, to the visual capabilities of dipterans.

One of the most remarkable results of this study is the enhanced conspicuousness of green–yellow relative to green flowers for both bees and flies. Given that no distinctive features of surface cells responsible for structural colours have been reported previously in green or green–yellow flowers (Kay *et al.*, 1981; Yuan *et al.*, 2023), we can infer that flower conspicuousness is influenced primarily by pigments accumulated in cells of floral advertising structures (Van Der Kooi *et al.*, 2019; van der Kooi, 2021). Except for rare green–bluish colours produced by highly decorated anthocyanins (Mizuno *et al.*, 2021), the green colour of flowers is produced primarily by the accumulation of chlorophylls (Vignolini *et al.*, 2012; Narbona *et al.*, 2021b; Yuan *et al.*, 2023). Indeed, the decrease from 550 to 650 nm and the dip at 670 nm observed in the reflectance spectra of green or green–yellow flowers align with the light absorption characteristics of chlorophylls, particularly chlorophyll *a* (Lee, 2007; Narbona *et al.*, 2021a). Likewise, the absence or very low reflectance between 400 and 500 nm in some green flowers, particularly in the green–yellow ones, is likely to be attributable to light absorption by carotenoids (Lee, 2007; Narbona *et al.*, 2021b), which typically co-occur with chlorophylls (Vignolini *et al.*, 2012; Narbona *et al.*, 2021b; Yuan *et al.*, 2023). We propose that the selective pressures favouring increased visibility to pollinators might operate by reducing chlorophyll concentrations and increasing carotenoid levels in green flowers, thereby distinguishing the green–yellow flowers from the leaf background. However, not all green flowers need to adopt this strategy to enhance their visibility to pollinators. The development of green–yellow pigmentation to enhance conspicuousness might be constrained by a combination of phylogenetic, ecological and evolutionary factors, as plants adopt diverse strategies to attract pollinators based on their specific ecological niches. Furthermore, pigment production involves a metabolic cost (Chalker‐Scott, 1999; Gould, 2004), which suggests that most angiosperms are likely to produce only the minimum necessary pigments in their flowers, presumably compensating with alternative strategies to attract pollinators.

### Less noticeable green flowers might display additional traits to attract pollinators

We identified several green-flowered species with low chromatic contrasts that appear less conspicuous to bees and flies. However, this does not necessarily mean that these subtler flowers go unnoticed by pollinators. Colour detection in the visual system of hymenopterans involves both chromatic and achromatic visual processing, with the use of each or their combination depending on the size and distance to a flower (Giurfa *et al.*, 1996; Dyer *et al.*, 2008; Ortiz *et al.*, 2021; van der Kooi and Kelber, 2022). Additionally, these factors can vary considerably depending on surrounding stimuli, lighting conditions or even environmental context, among others. As a result, the same physical stimulus might be interpreted as an entirely different colour, potentially influencing how bees respond to identical spectral cues (Garcia *et al.*, 2021; Lunau and Dyer, 2024; Shrestha *et al.*, 2024). In this study, species such as *Alchemilla alpina*, *Arum italicum* and most species of *Euphorbia* showed low chromatic contrasts while displaying high achromatic contrasts (Supplementary Data Fig. S4). High achromatic contrasts might benefit these less visually striking species by allowing honeybees and bumblebees to detect their flowers from long distances (Giurfa *et al.*, 1996; Spaethe *et al.*, 2001; Dyer *et al.*, 2008; but see Ng *et al.*, 2018). Green flowers might have other colour-related characteristics that enhance their attractiveness to pollinators, such as colour patterns, that improve flower detection by pollinators (De Ibarra *et al.*, 2015), as seen in *Nigella orientalis* (Yuan *et al.*, 2023). Likewise, green flowers can use alternative or complementary cues to lure bees or other insects. For instance, green-flowered species not included in this study, such as *Veratum album* or *Hedera helix*, produce a foul scent that attracts flies and ants (Faegri and van der Pijl, 1979; Kato *et al.*, 2009; Lukas *et al.*, 2020). Pollinator foraging decisions are influenced by a variety of factors, with colour being one of the most significant, although alternative or complementary cues are clearly important in flower identification. For instance, the significance of scent and colour can vary, shaped by the particular combination of these traits and the unique preferences of each bee (Chittka and Wells, 2004). Hence, other floral traits might enhance the detectability of flowers that are less prominent, such as the green ones in this study, making them more noticeable to pollinators.

## Conclusion

In conclusion, entomophilous green flowers have traditionally been considered invisible to pollinators, relying instead on scent cues for their attractiveness (Faegri and van der Pijl, 1979). Our results question this assertion, at least in part, because many species with green or green–yellow flowers can be perceived by both bees and flies. The combination of chlorophylls and carotenoids appears to play an important role in making green flowers visually more noticeable to pollinators (e.g. Martins *et al.*, 2021; Ortiz *et al.*, 2021). An intriguing question stemming from our study is why entomophilous green flowers persist instead of transitioning to green–yellow flowers, which we predict would be advantageous for pollinator detection. Although many green flowers can obtain a fraction of energy and carbon directly through photosynthesis within their tissues (Bazzaz *et al.*, 1979), the metabolic cost associated with acquiring new pigments (Chalker‐Scott, 1999; Gould, 2004), especially when alternative strategies to attract pollinators might be available, might have constrained the emergence of carotenoids in certain lineages.

## Supplementary Data

Supplementary data are available at *Annals of Botany* online and consist of the following.

Table S1: information about the 30 species with green or green–yellow flowers analysed in this study. Table S2: list of species used in this study along with their flower colour as perceived by the human eye. Figure S1: UV–VIS spectral reflectance of the 30 species that display green (A) or green–yellow (B) flowers included in this study. Figure S2: principal component analysis of the reflectance data from 30 species, grouped according to human visual perception into green (dark green circles) and green–yellow (lime green triangles) flowers. Figure S3: average UV–VIS spectral reflectance of typical blue–violet (*n* = 28), pink (*n* = 25), white (*n* = 9) and yellow (*n* = 38) flowers. Figure S4: achromatic contrast values of the 30 species that display green (dark green bars) or green–yellow (lime green bars) flowers, obtained in the pollinator visual systems of bees, are shown. Figure S5: violin plots with boxplots represent the distribution of achromatic contrast values, expressed in Euclidean distance units, obtained from the vision model of bees. Figure S6: spectra of the four species with green flowers that do not generate marker points.

mcae213_suppl_Supplementary_Table_S1

mcae213_suppl_Supplementary_Table_S2

mcae213_suppl_Supplementary_Figure_S1

mcae213_suppl_Supplementary_Figure_S2

mcae213_suppl_Supplementary_Figure_S3

mcae213_suppl_Supplementary_Figure_S4

mcae213_suppl_Supplementary_Figure_S5

mcae213_suppl_Supplementary_Figure_S6
