# Supplementary material for: Green flowers need yellow to get noticed in a green world
Source: Ann Bot. 2024 Dec 10;135(7):1281–92. doi: 10.1093/aob/mcae213 (PMC12358037; doi:10.1093/aob/mcae213)
Supplement: mcae213_suppl_Supplementary_Table_S1 [file mcae213_suppl_supplementary_table_s1.docx]

**Supplementary material**

**Table S1.** Information about the 30 species with green or green-yellow flowers analysed in this study: Taxonomical classification, flower colour to the human eye, flower colour category as perceived by each pollinator group considered in this study (hymenopterans and dipterans), location or FReD ID, number of reflectance spectra per species (N), floral structured from which the reflectance data were obtained (i.e., petal, spathe, flower, and bract), and main groups of pollinators visiting flowers according to the literature (DI = dipterans; HY = hymenopterans; CO = coleopterans; HE = hemipterans; LE = lepidopterans).

| **Family**  Species | | **Human colour** | | **Bee colour** | **Fly colour** | **Country/Province or FReD ID** | **N** | **Floral structure** | **Main group of pollinators** |
| --- | --- | --- | --- | --- | --- | --- | --- | --- | --- |
| **Amaryllidaceae** | |  | |  |  |  |  |  |  |
| *Narcissus viridiflorus* | | Green | | Blue-Green | Yellow | Spain / Cadiz | 8 | Petal | NA |
| **Araceae** | |  | |  |  |  |  |  |  |
| *Arum italicum* | | Green | | UV-Blue | Blue | Spain / Seville | 2 | Spathe | DI (Albre *et al.* 2003) |
| **Aristolochiaceae** | |  | |  |  |  |  |  |  |
| *Aristolochia paucinervis* | | Green | | UV | UV | Spain / Huelva | 3 | Petal | DI (Berjano *et al.* 2006) |
| **Asparagaceae** | |  | |  |  |  |  |  |  |
| *Asparagus horridus* | | Green | | Green | Yellow | Spain / Huelva | 3 | Petal | NA |
| **Asteraceae** | |  | |  |  |  |  |  |  |
| *Matricaria aurea* | | Green-yellow | | Green | Yellow | FReD (Israel; ID 2909) | 1 | Flower | HY, DI, CO (Armold *et al.* 2010) |
| *Matricaria discoidea* | | Green | | Green | Yellow | FReD (Austria; ID 1316) | 1 | Flower | HY (Gresty *et al.* 2018) |
| **Celastraceae** | |  | |  |  |  |  |  |  |
| *Euonymus europaeus* | | Green | | UV-Green | Purple | FReD (Germany; ID 1990) | 1 | Petal | DI, HY (Thomas *et al.* 2011) |
| **Euphorbiaceae** | |  | |  |  |  |  |  |  |
| *Euphorbia boetica* | | Green | | Green | Purple | Spain / Huelva | 4 | Bract | HY, DI, CO, HE (Narbona 2002) |
| *Euphorbia helioscopia* | | Green | | Green | Purple | Spain / Seville | 4 | Bract | HY, CO, HE (Kim and Park 2014) |
| *Euphorbia hierosolymitana* | | Green-yellow | | Green | Yellow | FReD (Israel; ID 2681) | 1 | Bract | DI (Armold *et al.* 2010) |
| *Euphorbia nicaeensis* | | Green | | Green | Purple | Spain / Cadiz | 3 | Bract | HY, DI, CO, HE (Narbona 2002) |
| *Euphorbia peplus* | | Green | | Green | Yellow | Spain / Seville | 1 | Bract | HY, DI (Asenbaum *et al.* 2021) |
| *Euphorbia segetalis* | | Green | | Green | Purple | Spain / Seville | 2 | Bract | HY, DI, CO (E. Narbona, unpubl. res.) |
| *Euphorbia serrata* | | Green | | Green | Purple | Spain / Cadiz | 3 | Bract | HY, DI (Guiller *et al.* 2016) |
| *Euphorbia terracina* | | Green | | Green | Yellow | Spain / Seville | 10 | Bract | HY, DI, CO (E. Narbona, unpubl. res.) |
| **Liliaceae** | |  | |  |  |  |  |  |  |
| *Paris quadrifolia* | | Green | | Green | Yellow | FReD (Germany; ID 1493) | 1 | Petal | DI (Jacquemyn and Brys 2008) |
| *Tofieldia calyculata* | | Green-yellow | | Green | Yellow | FReD (Austria; ID 1924) | 1 | Petal | NA |
| **Ranunculaceae** | |  | |  |  |  |  |  |  |
| *Helleborus foetidus* | | Green | | Blue-Green | Yellow | Spain / Cadiz | 3 | Petal | HY (Herrera *et al.* 2002) |
| **Rhamnaceae** | |  | |  |  |  |  |  |  |
| *Rhamnus lycioides* | | Green | | Green | Yellow | Spain / Cádiz | 3 | Petal | HY, DI (D. Pareja, unpubl. res.) |
| *Rhamnus oleoides* | | Green-yellow | | Green | Yellow | Spain / Cádiz | 3 | Petal | NA |
| **Rosaceae** | |  | |  |  |  |  |  |  |
| *Alchemilla alpina* | | Green | | Green | Yellow | FReD (Austria; ID 1806) | 1 | Petal | HY (Jervis *et al.* 1993) |
| *Alchemilla fissa* | | Green-yellow | | Green | Yellow | FReD (Austria; ID 1132) | 1 | Petal | NA |
| *Alchemilla glabra* | | Green-yellow | | Green | Yellow | FReD (Norway; ID 1133) | 1 | Petal | NA |
| *Alchemilla vulgaris* | | Green-yellow | | Green | Yellow | FReD (Austria; ID 1895) | 1 | Petal | NA |
| **Rubiaceae** | |  | |  |  |  |  |  |  |
| *Rubia peregrina* | | Green | | Blue-Green | Yellow | Spain / Seville | 5 | Petal | DI (D. Pareja, unpubl. Res.) |
| **Santalaceae** | |  | |  |  |  |  |  |  |
| *Osyris lanceolata* | | Green | | UV-Blue | UV | Spain / Cadiz | 1 | Petal | DI (Gulías and Traveset 2012) |
| *Viscum cruciatum* | | Green-yellow | | Green | Yellow | Spain / Cadiz | 3 | Petal | HY, DI (Aparicio *et al.* 1995) |
| **Saxifragaceae** | |  | |  |  |  |  |  |  |
| *Chrysosplenium alternifolium* | | Green-yellow | | Green | Purple | FReD (Austria; ID 3137) | 1 | Petal | NA |
| **Solanaceae** | |  | |  |  |  |  |  |  |
| *Withania frutescens* | | Green-yellow | | Green | Yellow | Spain / Almeria | 5 | Petal | NA |
| **Thymelaeaceae** | |  | |  |  |  |  |  |  |
| *Daphne laureola* | | Green-yellow | | Blue- Green | Yellow | Spain / Cadiz | 6 | Petal | HY, CO, LE (Alonso 2005) |
|  |  | | *NA = Information not available in the literature.* | | | | | | |

**References of Table S1.**

**Albre J, Quilichini A, Gibernau M. 2003.** Pollination ecology of *Arum italicum* (Araceae). *Botanical Journal of the Linnean Society* **141**: 205–214.

**Alonso C. 2005.** Pollination success across an elevation and sex ratio gradient in gynodioecious *Daphne laureola*. *American Journal of Botany* **92**: 1264–1269.

**Aparicio A, Gallego MJ, Vazquezt C. 1995.** Reproductive Biology of *Viscum cruciatum* (Viscaceae) in Southern Spain. *International Journal of Plant Sciences* **156**: 42–49.

**Armold SEJ, Faruq S, Savolainen V, McOwan PW, Chittka L. 2010.** FReD: The floral reflectance database - a web portal for analyses of flower colour. *PLoS ONE* **5**: e14287.

**Asenbaum J, Schäffler I, Etl F, Dötterl S, Schönenberger J, Chartier M. 2021.** Comparative pollination ecology of five European *Euphorbia* Species. *International Journal of Plant Sciences* **182**: 763–777.

**Berjano R, De Vega C, Arista M, Ortiz PL, Talavera S. 2006.** A multi-year study of factors affecting fruit production in *Aristolochia paucinervis* (Aristolochiaceae). *American Journal of Botany* **93**: 599–606.

**Gresty CEA, Clare E, Devey DS, et al. 2018.** Flower preferences and pollen transport networks for cavity-nesting solitary bees: Implications for the design of agri-environment schemes. *Ecology and Evolution* **8**: 7574–7587.

**Guiller C, Affre L, Albert CH, Tatoni T, Dumas E. 2016.** How do field margins contribute to the functional connectivity of insect-pollinated plants? *Landscape Ecology* **31**: 1747–1761.

**Gulías J, Traveset A. 2012.** Altitudinal variation in the reproductive performance of the Mediterranean shrub *Rhamnus lycioides* L. *Journal of Plant Ecology* **5**: 330–336.

**Herrera CM, Cerdá X, García MB, et al. 2002.** Floral integration, phenotypic covariance structure and pollinator variation in bumblebee-pollinated *Helleborus foetidus*. *Journal of Evolutionary Biology* **15**: 108–121.

**Jacquemyn H, Brys R. 2008.** Density-dependent mating and reproductive assurance in the temperate forest herb *Paris quadrifolia* (Trilliaceae). *American Journal of Botany* **95**: 294–298.

**Jervis MA, Kidd NAC, Fitton MG, Huddleston T, Dawah HA. 1993.** Flower-visiting by hymenopteran parasitoids. *Journal of natural history* **27**: 67-67.

**Kim D Il, Park KR. 2014.** Pollination study of *Euphorbia helioscopia* (Euphorbiaceae). *Korean Journal of Plant Taxonomy* **44**: 281–287.

**Narbona E. 2002.** Estrategias reproductivas de dos especies perennes de *Euphorbia*. PhD Thesis, University of Seville, Spain.

**Thomas PA, El-Barghathi M, Polwart A. 2011.** Biological Flora of the British Isles: *Euonymus europaeus* L. *Journal of Ecology* **99**: 345–365.
