# Supplementary material for: Green flowers need yellow to get noticed in a green world
Source: Ann Bot. 2024 Dec 10;135(7):1281–92. doi: 10.1093/aob/mcae213 (PMC12358037; doi:10.1093/aob/mcae213)
Supplement: mcae213_suppl_Supplementary_Table_S2 [file mcae213_suppl_supplementary_table_s2.docx]

**Supplementary material**

**Table S2.** List of species used in this study along with their flower colour as perceived by the human eye. For the bee and fly visual models, values of excitation of the photoreceptors (E_UV_, E_B_, and E_G_ for bees; R7p, R7y, R8p, and R8y for flies), coordinates and section occupied in each visual model, and chromatic and achromatic contrasts (the latter, only for bees) are provided for each species.

|  |  |  | **Bee visual model** | | | | | | | |  | **Fly visual model** | | | | | | | |
| --- | --- | --- | --- | --- | --- | --- | --- | --- | --- | --- | --- | --- | --- | --- | --- | --- | --- | --- | --- |
| **Species** | **Colour** |  | **E_UV_** | **E_B_** | **E_G_** | **x** | **y** | **Section** | **Chromatic contrast** | **Achromatic contrast** |  | **R7p** | **R7y** | **R8p** | **R8y** | **x** | **y** | **Section** | **Chromatic contrast** |
| *Alchemilla alpina* | G |  | 0.6657 | 0.6642 | 0.7117 | 0.0398 | -0.0244 | Green | 0.0467 | 0.2117 |  | 0.2395 | 0.2320 | 0.2501 | 0.2784 | -0.0106 | -0.0465 | Yellow | 0.0477 |
| *Aristolochia paucinervis* | G |  | 0.6547 | 0.5976 | 0.6083 | -0.0402 | -0.0339 | UV | 0.0526 | 0.1083 |  | 0.2985 | 0.2488 | 0.2229 | 0.2298 | 0.0757 | 0.0189 | UV | 0.0780 |
| *Arum italicum* | G |  | 0.7653 | 0.7736 | 0.7166 | -0.0422 | 0.0326 | UV-Blue | 0.0533 | 0.2166 |  | 0.2551 | 0.2784 | 0.2648 | 0.2017 | -0.0098 | 0.0768 | Blue | 0.0774 |
| *Asparagus horridus* | G |  | 0.3832 | 0.4199 | 0.5362 | 0.1326 | -0.0398 | Green | 0.1384 | 0.0362 |  | 0.1933 | 0.2093 | 0.2562 | 0.3412 | -0.0629 | -0.1319 | Yellow | 0.1462 |
| *Euonymus europaeus* | G |  | 0.6288 | 0.5888 | 0.6132 | -0.0135 | -0.0323 | UV-Green | 0.0350 | 0.1132 |  | 0.2725 | 0.2461 | 0.2312 | 0.2502 | 0.0412 | -0.0041 | Purple | 0.0414 |
| *Euphorbia boetica* | G |  | 0.6280 | 0.5785 | 0.6919 | 0.0554 | -0.0814 | Green | 0.0985 | 0.1919 |  | 0.2516 | 0.2139 | 0.2165 | 0.3180 | 0.0350 | -0.1041 | Purple | 0.1099 |
| *Euphorbia helioscopia* | G |  | 0.7154 | 0.6693 | 0.7965 | 0.0702 | -0.0867 | Green | 0.1115 | 0.2965 |  | 0.2390 | 0.1997 | 0.2225 | 0.3388 | 0.0166 | -0.1392 | Purple | 0.1401 |
| *Euphorbia nicaeensis* | G |  | 0.7546 | 0.7260 | 0.8217 | 0.0581 | -0.0621 | Green | 0.0851 | 0.3217 |  | 0.2386 | 0.2074 | 0.2346 | 0.3194 | 0.0039 | -0.1121 | Purple | 0.1121 |
| *Euphorbia peplus* | G |  | 0.6513 | 0.6868 | 0.7253 | 0.0640 | -0.0015 | Green | 0.0640 | 0.2253 |  | 0.2115 | 0.2319 | 0.2672 | 0.2894 | -0.0557 | -0.0575 | Yellow | 0.0801 |
| *Euphorbia segetalis* | G |  | 0.7189 | 0.6713 | 0.7809 | 0.0537 | -0.0786 | Green | 0.0952 | 0.2809 |  | 0.2506 | 0.2103 | 0.2163 | 0.3229 | 0.0343 | -0.1126 | Purple | 0.1177 |
| *Euphorbia serrata* | G |  | 0.6890 | 0.6217 | 0.7735 | 0.0732 | -0.1096 | Green | 0.1318 | 0.2735 |  | 0.2478 | 0.2020 | 0.2020 | 0.3482 | 0.0459 | -0.1463 | Purple | 0.1533 |
| *Euphorbia terracina* | G |  | 0.7013 | 0.6805 | 0.7879 | 0.0750 | -0.0641 | Green | 0.0987 | 0.2879 |  | 0.2299 | 0.2061 | 0.2304 | 0.3337 | -0.0005 | -0.1276 | Yellow | 0.1276 |
| *Helleborus foetidus* | G |  | 0.2357 | 0.4300 | 0.6255 | 0.3375 | -0.0006 | Green | 0.3375 | 0.1255 |  | 0.0865 | 0.1712 | 0.2786 | 0.4637 | -0.1921 | -0.2924 | Yellow | 0.3499 |
| *Matricaria discoidea* | G |  | 0.5438 | 0.5635 | 0.6144 | 0.0612 | -0.0156 | Green | 0.0631 | 0.1144 |  | 0.2173 | 0.2405 | 0.2508 | 0.2915 | -0.0335 | -0.0510 | Yellow | 0.0610 |
| *Narcissus viridiflorus* | G |  | 0.5363 | 0.6846 | 0.6704 | 0.1161 | 0.0812 | Blue-Green | 0.1417 | 0.1704 |  | 0.1535 | 0.2474 | 0.3212 | 0.2779 | -0.1676 | -0.0305 | Yellow | 0.1704 |
| *Osyris lanceolata* | G |  | 0.6315 | 0.6219 | 0.5706 | -0.0528 | 0.0208 | UV-Blue | 0.0567 | 0.0706 |  | 0.2658 | 0.2751 | 0.2497 | 0.2093 | 0.0161 | 0.0658 | UV | 0.0678 |
| *Paris quadrofolia* | G |  | 0.2545 | 0.3221 | 0.4595 | 0.1776 | -0.0349 | Green | 0.1810 | 0.0405 |  | 0.1583 | 0.2032 | 0.2555 | 0.3830 | -0.0972 | -0.1798 | Yellow | 0.2044 |
| *Rhamnus lycioides* | G |  | 0.5119 | 0.5003 | 0.6208 | 0.0943 | -0.0661 | Green | 0.1151 | 0.1208 |  | 0.2226 | 0.2101 | 0.2381 | 0.3291 | -0.0155 | -0.1190 | Yellow | 0.1200 |
| *Rubia peregrina* | G |  | 0.4843 | 0.5694 | 0.6381 | 0.1332 | 0.0082 | Blue-Green | 0.1335 | 0.1381 |  | 0.1733 | 0.2249 | 0.2785 | 0.3233 | -0.1052 | -0.0984 | Yellow | 0.1440 |
| *Alchemilla fissa* | G-Y |  | 0.3476 | 0.3837 | 0.5474 | 0.1730 | -0.0638 | Green | 0.1843 | 0.0474 |  | 0.1798 | 0.1942 | 0.2456 | 0.3804 | -0.0658 | -0.1862 | Yellow | 0.1975 |
| *Alchemilla glabra* | G-Y |  | 0.1827 | 0.3049 | 0.5349 | 0.3050 | -0.0539 | Green | 0.3097 | 0.0349 |  | 0.1136 | 0.1584 | 0.2661 | 0.4619 | -0.1526 | -0.3035 | Yellow | 0.3397 |
| *Alchemilla vulgaris* | G-Y |  | 0.2607 | 0.3664 | 0.6091 | 0.3017 | -0.0685 | Green | 0.3094 | 0.1091 |  | 0.1205 | 0.1642 | 0.2497 | 0.4657 | -0.1293 | -0.3015 | Yellow | 0.3280 |
| *Chrysosplenium alternifolium* | G-Y |  | 0.3783 | 0.3278 | 0.5831 | 0.1773 | -0.1529 | Green | 0.2341 | 0.0831 |  | 0.2120 | 0.1749 | 0.1977 | 0.4154 | 0.0143 | -0.2404 | Purple | 0.2408 |
| *Daphne laureola* | G-Y |  | 0.1701 | 0.4653 | 0.6564 | 0.4211 | 0.0520 | Blue-Green | 0.4243 | 0.1564 |  | 0.0614 | 0.1644 | 0.3016 | 0.4726 | -0.2402 | -0.3082 | Yellow | 0.3907 |
| *Euphorbia hierosolymitana* | G-Y |  | 0.4363 | 0.4144 | 0.6831 | 0.2137 | -0.1453 | Green | 0.2584 | 0.1831 |  | 0.1878 | 0.1718 | 0.2053 | 0.4351 | -0.0174 | -0.2633 | Yellow | 0.2639 |
| *Matricaria aurea* | G-Y |  | 0.1679 | 0.1860 | 0.5911 | 0.3665 | -0.1935 | Green | 0.4144 | 0.0911 |  | 0.1200 | 0.1195 | 0.1935 | 0.5669 | -0.0735 | -0.4474 | Yellow | 0.4534 |
| *Rhamnus oleoides* | G-Y |  | 0.1202 | 0.2560 | 0.5014 | 0.3302 | -0.0548 | Green | 0.3347 | 0.0014 |  | 0.0939 | 0.1458 | 0.2693 | 0.4910 | -0.1755 | -0.3452 | Yellow | 0.3872 |
| *Tofieldia calyculata* | G-Y |  | 0.4671 | 0.5649 | 0.6997 | 0.2014 | -0.0185 | Green | 0.2023 | 0.1997 |  | 0.1573 | 0.1968 | 0.2695 | 0.3763 | -0.1122 | -0.1795 | Yellow | 0.2117 |
| *Viscum cruciatum* | G-Y |  | 0.0940 | 0.2650 | 0.4833 | 0.3372 | -0.0237 | Green | 0.3380 | 0.0167 |  | 0.0760 | 0.1546 | 0.2866 | 0.4828 | -0.2105 | -0.3281 | Yellow | 0.3899 |
| *Withania frutescens* | G-Y |  | 0.4443 | 0.5642 | 0.6948 | 0.2169 | -0.0053 | Green | 0.2170 | 0.1948 |  | 0.1298 | 0.2082 | 0.2701 | 0.3919 | -0.1403 | -0.1837 | Yellow | 0.2311 |
